# Highly Uniform Spherical MoO_2_-MoO_3_/Polypyrrole Core-Shell Nanocomposite as an Optoelectronic Photodetector in UV, Vis, and IR Domains

**DOI:** 10.3390/mi14091694

**Published:** 2023-08-30

**Authors:** Asmaa M. Elsayed, Fatemah H. Alkallas, Amira Ben Gouider Trabelsi, Mohamed Rabia

**Affiliations:** 1 TH-PPM Group, Physics Department, Faculty of Science, Beni-Suef University, Beni-Suef 62514, Egypt; asmaa.elsayed@science.bsu.edu.eg; 2 Department of Physics, College of Science, Princess Nourah bint Abdulrahman University, P.O. Box 84428, Riyadh 11671, Saudi Arabia; fhalkallas@pnu.edu.sa; 3 Nanomaterials Science Research Laboratory, Chemistry Department, Faculty of Science, Beni-Suef University, Beni-Suef 62514, Egypt

**Keywords:** optoelectronic, photodetector, nanocomposite, MoO_2_, polypyrrole, MoO_3_

## Abstract

A highly uniform spherical MoO_2_-MoO_3_/polypyrrole core-shell nanocomposite has been successfully synthesized as an optoelectronic photon sensing material, capable of detecting light in the UV, Vis, and IR domains. The nanocomposite is prepared through the oxidation of pyrrole using Na_2_MoO_4_, resulting in a uniform spherical morphology that has been confirmed by TEM, theoretical modeling, and SEM analyses. This morphology contributes to its promising optical behavior, characterized by a small bandgap of 1.36 eV. The optoelectronic photosensing capability of the nanocomposite has been evaluated across the UV, Vis, and IR spectra, demonstrating high efficiency. The photoresponsivity R values indicate the ability of the nanocomposite to generate hot electrons in response to incident photons. With an R value of 4.15 mA·W^−1^ at 440 nm, this optoelectronic device exhibits considerable promise for integration into an advanced technological apparatus. The detection (D) value of 9.30 × 10^8^ Jones at 440 nm further confirms the high sensitivity in the Vis region. The excellent stability of the device can be attributed to the inherent MoO_2_-MoO_3_ oxide and Ppy polymer materials. This stability has been demonstrated through reproducibility studies and current-voltage measurements under various optical conditions. The combination of stability, efficiency, and sensitivity makes this optoelectronic device well suited for light sensing applications in both industrial and commercial settings. Its promising performance opens up opportunities for advancements in various fields requiring accurate and reliable light detection.

## 1. Introduction

Light plays a crucial role as an energy source in our daily lives, offering heat and vision to creatures, where both natural and artificial photosynthesis reactions highly depend on light. Optoelectronic devices have become a vital component in modern technology for several applications like smart windows and aircraft [1,2,3].

Photodetectors’ work principally depends on their ability to detect light with various frequency. Indeed, activated photons received through the surface generate current density (J_ph_) values [4,5]. The latter is commonly stopped on the intensity of the photon flux for semiconductors. This is assigned to the large photon flux liberating more electrons at the surface illustrating the resonance motion J_ph_ values that measure the rate of liberated electrons imaging the photodetector sensitivity to the light through photoresponsivity (R) and detectivity (D) [6,7].

Inorganic materials are widely exploited as photodetectors due to their ability to detect light through accepting photon flux in their active sites. Last century, scientists tried to increase the surface area of these materials by increasing their active sites. This has been established through the change in the specific geometry of the material into nanorods, nanowires, nanotubes, and nanosheets. Wang et al. demonstrate the high surface area obtained for CuO nanowires where a good light detection has been located in the IR domain. Nevertheless, a small J_ph_ (20 µA) at a high application has been obtained. These studies open doors for using inorganic oxide materials for light detection and sensing. Bai et al. [8] reported a slight enhancement in J_ph_ values (107 µA) while incorporating ZnO in CuO. Also, previous studies demonstrate the low efficiency of the TiO_2_ photodetector that did not reach 1% [9]. 

These hampering faces while used organic material raise interest to polymer materials applications as based material for photodetectors. This is owing to their stability, composite contacts, exceptional sensitivity cost effective, mass production, and easy preparation [10,11,12,13]. In fact, various polymers have been utilized as photodetectors-based material like poly-3-hexylthiophene with its derivatives or polypyrrole. Nonetheless, these studies resulted in low J_ph_ values with only 0.01 mA at 2 V like that produced for PMMA/styrene/carbon nanotube. Similarly, theoretical studies involving aniline derivatives, such as benzodithiophene/fluorine, showed amelioration of the optical properties. Mainly, earlier studies utilizing polymers as based material for photodetectors demonstrate several drawbacks like the low J_ph_ values ranging from 0.001 to 0.1 mA·cm^−2^, low reproducibility, and limited sensitivity to specific regions of the electromagnetic spectrum, such as UV, Vis, or IR [14,15].

Atta and co-authors conducted investigations involving polypyrrole composites, initially incorporating graphene oxide and subsequently integrating NiO. The primary focus of their research was to amplify the optical performance of these composite structures, with the ultimate goal of advocating for their application within the realm of optoelectronics. This recommendation was predicated on the notable augmentation of optical properties exhibited by these materials, which were further substantiated by their inherent semiconducting characteristics [16,17].

In this work, we investigated the current to address the limitations seen so far for polymer-based optoelectronic devices. We synthesized a photodetector using a combination of MoO_2_-MoO_3_, and Ppy, which resulted in activation of the surface sites. This photodetector was able to detect light in the UV, Vis, and NIR regions and exhibited good responsivity and detectivity values. The device also showed high reproducibility and responded well to an on/off chop light.

## 2. Experimental Part

### 2.1. Materials

Sodium molybdate (Na_2_MoO_4_) and pyrrole (high purity, 999%) were sourced from Winlab (Watford, UK) and Sigma Aldrich Co., Ltd. (Tokyo, Japan) in UK and Japan, respectively, while dimethylformamide (DMF, (high purity, 999%) was acquired in the USA, Sigma Aldrich (St. Louis, MO, USA). HCl (37%) was sourced from Germany (Merck Co., Ltd., Darmstadt, Germany). K_2_S_2_O_8_ was supported from Egypt (Pio-Chem Co., Ltd., Giza, Egypt). 

### 2.2. Ppy Preparation

The pyrrole undergoes oxidative polymerization through radical formation and subsequent connection of these monomers to form Ppy using 0.15 M K_2_S_2_O_8_ as an oxidant. The monomer is dissolved in 0.5 M HCl and stirred for 15 min, while the oxidant is dissolved in distilled water. The addition of the oxidant initiates polymerization, which continues for 24 h. On the second day, the polymer is collected and treated with distilled water to remove any surface contamination.

### 2.3. MoO_2_-MoO_3_/Polypyrrole Core-Shell Thin Film Photodetector 

The MoO_2_-MoO_3_/Ppy core-shell nanocomposite is formed through a one-pot reaction and by using Na_2_MoO_4_ oxidant to generate the pyrrole free radical, which then incorporates MoO_2_ and MoO_3_ inorganic nanomaterials into the polymer chains. This reaction is similar to the oxidative polymerization used for Ppy formation. The same concentration of HCl is used to dissolve the pyrrole monomer, and a glass slide is added to the beaker during polymerization to form a MoO_2_-MoO_3_/Ppy thin film. The molar ration of the oxidant to the monomer is 2.5:1, in total volume of 100 mL. The MoO_2_-MoO_3_/Ppy thin film is collected the following day and cleaned using distilled water to remove any surface contaminants.

### 2.4. The Electrical Measurements for the Constructed MoO_2_-MoO_3_/Ppy Core-Shell Thin Film Photodetector

**Figure 1:**
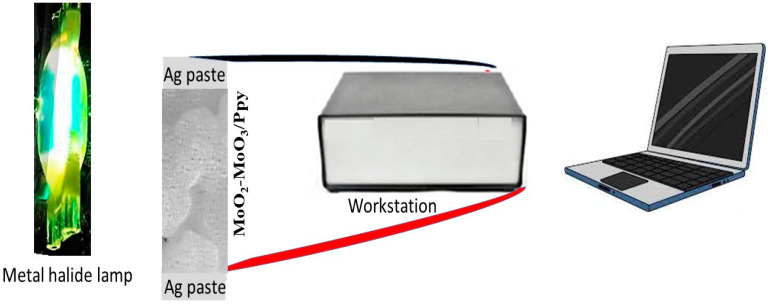
Schematic diagram of electrical measurements of the MoO_2_-MoO_3_/Ppy thin film photodetector (area, 1.0 cm^2^) through the CHI608E workstation and under a metal halide lamp (100 mW/cm^2^).

The electrical measurements of the MoO_2_-MoO_3_/Ppy photodetector were demonstrated using a CHI608E workstation, and the schematic diagram in Figure 1 was followed for the connection method. The metal halide lamp was directed towards the thin film nanocomposite to illuminate it, and the resulting photocurrent (J_ph_) was measured directly using the connected computer. The dark current (J_o_) was also measured to determine the normal conductivity of the nanocomposite. The responsivity of the device, which measures the efficiency of converting incident light into photocurrent, was calculated for a wide range of wavelengths (340 to 730 nm), and values for R, LDR, and D were obtained.

## 3. Results and Discussion

### 3.1. Analyses Processes

**Figure 2:**
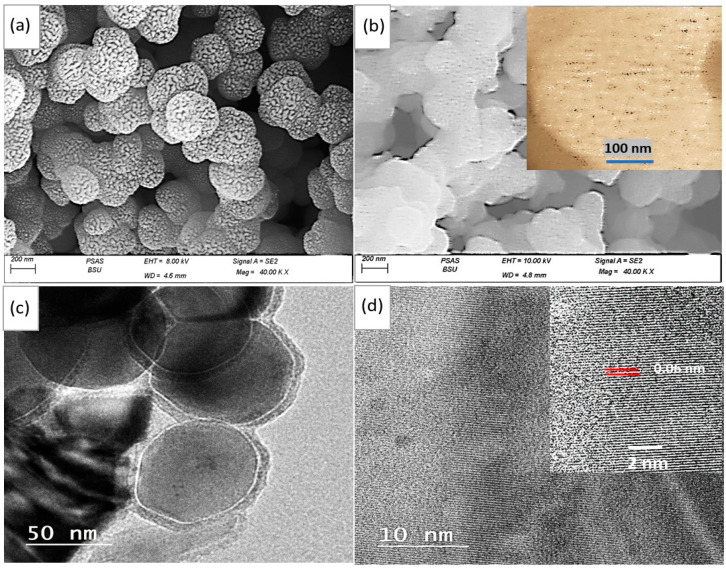
The SEM surface morphology of Ppy (**a**). The surface topography and morphology of the MoO_2_-MoO_3_/Ppy core-shell nanocomposite: (**b**) SEM and (**c**,**d**) TEM at various magnification scalebar.

The SEM images in Figure 2a,b confirm the particle distribution and surface behavior of both Ppy and MoO_2_-MoO_3_/Ppy. The surface behavior differs significantly before and after composite formation. The pure Ppy polymer shows porous spherical particles with a particle size range of 250 nm, where each large particle is made up of interconnected smaller particles. In contrast, the MoO_2_-MoO_3_/Ppy nanocomposite exhibits similar features with large spherical particles (200 nm) formed from smaller ones. However, the large particles in the nanocomposite are interconnected to form a network within the polymer composite. This network formation is a desirable feature for creating a thin film on glass, which can readily interact with light photons for photoelectrode applications.

A clear view of the morphological features of the MoO_2_-MoO_3_/Ppy nanocomposite is illustrated through TEM analyses (Figure 2c). The individual spherical core-shell structure of the nanocomposite is clearly visible with a core size of 73 nm and a shell thickness of 6 nm. This unique morphology is a crucial factor in determining the excellent optical behavior of the nanocomposite, as the thin shell allows for efficient electron transfer between the inside and outside of the core. At TEM high magnification (Figure 2d), the distance between adjacent layers appears well for the nanocomposite with an interlaminar space distance of 0.06 nm. The great layer order of this nanocomposite gives a great indication for a high and promising optical behavior [11,12]. 

**Figure 3:**
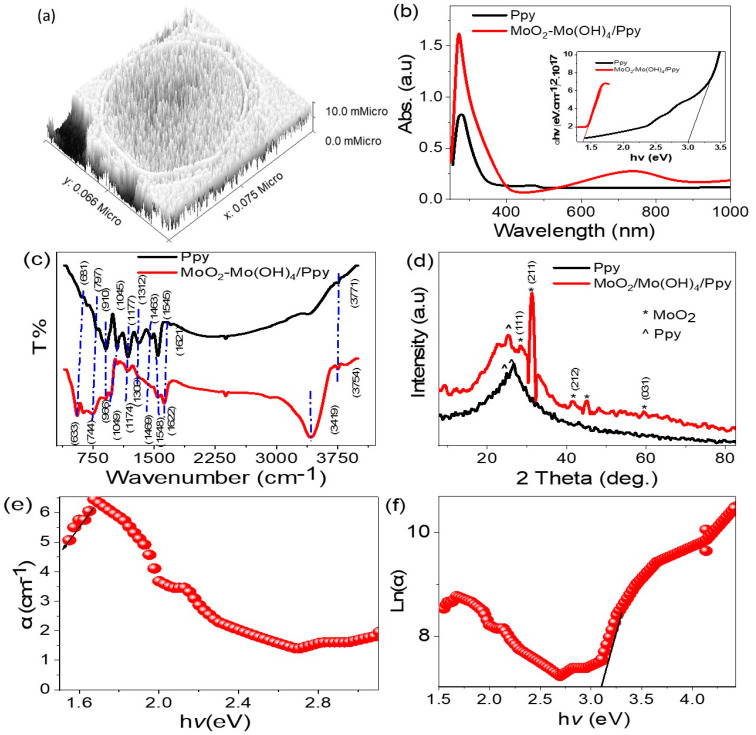
(**a**) The theoretical morphological of the MoO_2_-MoO_3_/Ppy core-shell nanocomposite. (**b**) The optical behavior, (**c**) FTIR, and (**d**) XRD pattern of Ppy and MoO_2_-MoO_3_/Ppy core-shell nanocomposite. (**e**,**f**) The absorption coefficient and Urbach energy, respectively, for the MoO_2_-MoO_3_/Ppy core-shell nanocomposite.

Figure 3a depicts the theoretical model of the MoO_2_-MoO_3_/Ppy core-shell nanocomposite, which was generated using the Gwydion program [18]. The image shows the surface roughness and particle size, with the particle consisting of a core and shell with sizes of 72 and 6 nm, respectively. The particle is formed by the agglomeration of small particles, ultimately leading to the formation of this large particle.

Figure 3b presents the evaluation of the optical behavior of the MoO_2_-MoO_3_/Ppy core-shell nanocomposite in comparison to the pure polymer, Ppy. This composite has significantly better optical properties than Ppy. While Ppy exhibits only one peak in the UV region, the composite displays two peaks: the first in the UV and visible region (ranging from 250 to 420 nm), and the second extending from the visible to the infrared region (ranging from 500 to 900 nm). This confirms the enhanced photoelectrode properties of the nanocomposite, which can potentially be utilized in various applications.

The bandgap value for the MoO_2_-MoO_3_/Ppy core-shell nanocomposite is estimated to be 1.36 eV, which is determined from the Tauc equation [19,20,21] as shown in the inserted figure. This value is considered an ideal bandgap for photocatalytic applications, suggesting the potential of this nanocomposite for such applications.

To provide a more comprehensive understanding of the optical properties of the MoO_2_-MoO_3_/Ppy core-shell nanocomposite, additional calculations were conducted. The absorption coefficient (α) and Urbach energy (E_U_) are estimated, and the results are depicted in Figure 3e,f, respectively. The calculated values for α and E_U_ are approximately 1.08 and 3 eV, respectively. The determination of α involved utilizing the absorbance (A) and film thickness (t) values, which were evaluated using Equation (1) [16,17]. On the other hand, E_U_ was derived using Equation (2) [16,17], which characterizes the energy tailing behavior as a function of the logarithmic value of α. These parameters offer valuable insights into the optical behavior and great contact between the MoO_2_-MoO_3_/Ppy core-shell nanocomposite.

(1) $$\alpha =\frac{2.303A}{t}$$

(2) $$\alpha =\alpha_{o}e^{h\nu /E_{U}}$$

These groups are analyzing the electron vibration using FTIR, as shown in Figure 3c. For Ppy, the main functional groups, such as C–N [22], C–H, C=C (benzene), and C=C (quinoid), were found at 1318, 1151, 1466, and 1622 cm^−1^, correspondingly. After the formation of the MoO_2_-MoO_3_/Ppy core-shell nanocomposite, the same functional groups were detected, but with shifts in the band locations due to the incorporation of MoO_2_-MoO_3_. These shifts can be either blue or red, depending on the inorganic material insertion on the electron vibration [23,24,25,26]. Table 1 summarizes the positions of these functional groups for Ppy and MoO_2_-MoO_3_/Ppy.

The XRD pattern shown in Figure 3d determines the crystalline behavior and size of the MoO_2_-MoO_3_/Ppy core-shell nanocomposite. Analysis of the XRD pattern revealed the presence of MoO_2_ and MoO_3_ inorganic materials within the polymer matrix. The peaks observed at 28.3°, 36.5°, 41.5°, and 59.4° is for the (111), (211), (212), and (031), respectively, of MoO_2_ (JCBDS 65-5787) [27,28]. The peaks observed at 31.3°, 39.7°, 45.2°, and 48.2° correspond to the (111), (040), (200), and (220) growth directions, respectively, of MoO_3_ (JCPDS-05-0508) [23,24]. This information provides insight into the crystalline structure of the nanocomposite and its potential properties.

The XRD pattern analysis also confirmed the crystalline behavior of Ppy in both the pure form and in the nanocomposite. In the pure Ppy sample (black curve), two crystalline peaks are observed at 24.8° and 27.0°. In the nanocomposite (red curve), these two peaks are also observed at 22.7° and 25.0°, respectively, with the appearance of a third peak located at 10°. The size of crystals in the nanocomposite was estimated using the Scherrer Equation (3) [25], with a calculated size of 69 nm. The value of λ and the full width half maximum were considered as the main parameters for estimating this size.

D = 0.9λ/W cosθ(3)

**Figure 4:**
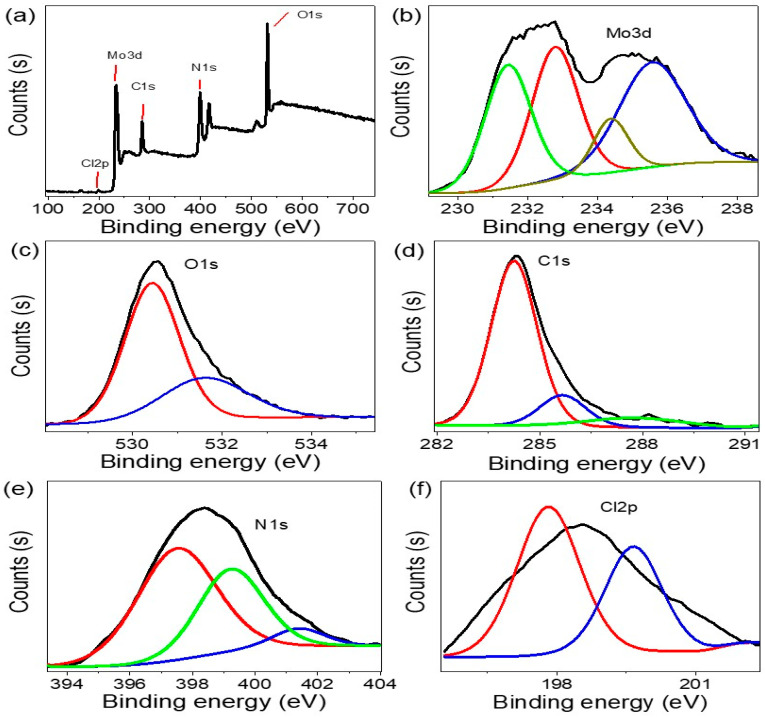
The MoO_2_-MoO_3_/Ppy XPS analysis: (**a**) survey, (**b**) Mo, (**c**) O, (**d**) C, (**e**) N, and (**f**) acid Cl spectra.

XPS determines the elemental behavior of the MoO_2_-MoO_3_/Ppy films that were grown (see, Figure 4). The XPS survey spectrum revealed the presence of several elements, including Mo, C, N, O, and Cl (see, Figure 4b–f). Specifically, the Mo3d spectrum displayed two prominent peaks located at 232.8 and 235 eV are for Mo 3d_3/2_ of MoO_2_. Additionally, a peak at approximately 235.8 eV, corresponding to Mo^6+^ 3d_5/2_ of MoO_3_, indicated significant oxidation of the Mo species.

The oxidation of Mo was further corroborated by the identification of the oxygen peak (O1s). The C1s, N1s, and O1s peaks were situated at energies of 284.5, 398, and 530.5 eV, respectively. These observations can be results from the p–p interaction in PPy component and the surrounding elements.

Overall, XPS analysis confirmed the presence of Mo, C, N, O, and Cl in the MoO_2_-MoO_3_/Ppy films. Moreover, the p–p interaction between PPy and the other elements was evidenced by the C1s, N1s, and O1s peak positions. These findings provide valuable insights into the elemental composition and chemical interactions within the MoO_2_-MoO_3_/Ppy films.

### 3.2. Electrical Testing

The light sensitivity of the MoO_2_-MoO_3_/Ppy core-shell nanocomposite is studied by the CHI608E device. The J_ph_ values obtained from these measurements indicate hot electrons generated from these promising materials under exposure to light, reflecting its light sensitivity. The photogenerated electrons observed in the MoO_2_-MoO_3_/Ppy core-shell nanocomposite are generated through the process of electron transfer between the bands. These transferred electrons are responsible for the generation of the J_ph_ observed in the electrical signal measurements.

The optoelectronic photoelectrode consisting of MoO_2_, MoO_3_, and Ppy exhibits a diverse light absorbance behavior, allowing it to capture photons across the electromagnetic spectrum from ultraviolet (UV) to infrared (IR). This characteristic is highly promising as it enables the generation of hot electrons and reflects in the observed current density (J_ph_) values. The optical bandgap of 1.36 eV further supports this behavior, indicating the efficient absorption of photons in this composite material. 

**Figure 5:**
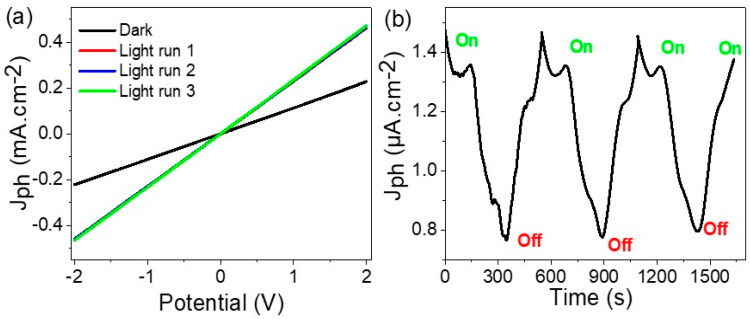
(**a**) The electrical testing of MoO_2_-MoO_3_/Ppy core-shell nanocomposite optoelectronic photoelectrode through the (**a**) potential-current in a voltage range −2.0 to +2.0 V and light illumination of 100 mW/cm^2^ and (**b**) time-current relations under dark and light illumination at bias voltage of 0.1 V and light illumination of 100 mW/cm^2^.

The behavior of the MoO_2_-MoO_3_/Ppy core-shell nanocomposite under light illumination can be effectively observed through the current-voltage, as indicated in Figure 5a. When the material is exposed to light, the generation of electrons occurs, resulting in their accumulation on the surface of the optoelectronic photoelectrode. However, under dark conditions, this process does not take place, resulting in J_ph_ (light-induced current density) and J_o_ (dark current density) measurements of 0.46 and 0.21 mA·cm^−2^, respectively.

The optoelectronic photoelectrode materials, including the inorganic components MoO_2_ and MoO_3_, as well as the highly stable Ppy, exhibit excellent stability and respond consistently to photons with stable current runs. This stability is evident from the similarity in values observed in runs 1 to 3.

In the reproducibility behavior analysis shown in Figure 5b, the difference values between J_ph_ (current density under light) and Jo (dark current density) are clearly observed. The values of 1.4 and 0.8 µA·cm^−2^ represent the behavior of the optoelectronic photoelectrode in light conditions, correspondingly. The excellent repeatability of these values highlights the stability of the photoelectrode.

Based on this stability and reproducibility, the MoO_2_-MoO_3_/Ppy core-shell nanocomposite optoelectronic photoelectrode holds great potential for application in advanced technology devices that require light sensing capabilities [15,19,20,21]. 

The sensitivity of the MoO_2_-MoO_3_/Ppy core-shell nanocomposite optoelectronic photoelectrode can be effectively tested by controlling the wavelength of the incident photons using optical filters. The energy of the generated hot electrons is dependent on the wavelength of the illuminating light, as each photon carries a specific energy determined by its wavelength. By employing different optical filters, the resulting J_ph_ values will vary, reflecting the efficiency of the photoelectrode in converting light energy into electrical current. This approach allows for a comprehensive evaluation of the photoelectrode’s sensitivity across different wavelength ranges.

**Figure 6:**
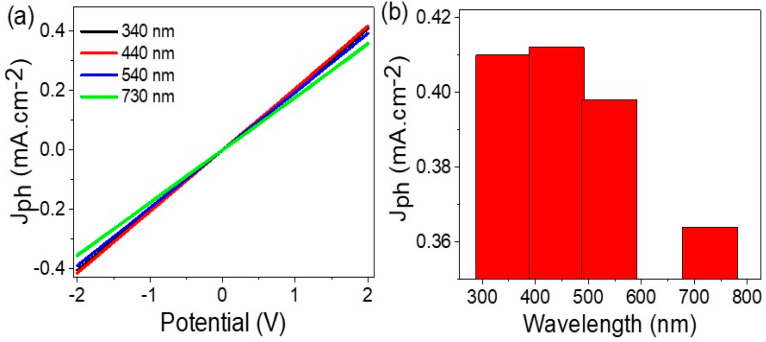
(**a**) The electrical testing of MoO_2_-MoO_3_/Ppy core-shell nanocomposite optoelectronic photoelectrode (1.0 cm^2^) through the (**a**) potential-current under various optical wavelength filters and (**b**) the J_ph_ at 2.0 V produced from the potential-current relation.

Figure 6a illustrates the current-potential relationship for different optical filters, showcasing the varied behavior of the MoO_2_-MoO_3_/Ppy core-shell nanocomposite photoelectrode. The J_ph_ values obtained at specific wavelengths are 0.41 mA·cm^−2^ at 440 nm and 0.36 mA·cm^−2^ at 730 nm. These values indicate the sensitivity of the photoelectrode to photons across a range of wavelengths. The summarized representation in Figure 6b further emphasizes the diverse response of the photoelectrode to different incident photons, highlighting its high sensitivity.

The R values [26] that estimate the conversion of photons into hot electrons of the MoO_2_-MoO_3_/Ppy core-shell nanocomposite optoelectronic photoelectrode is evaluated depending on the generated number of hot electrons. Through the calculation of photoresponsivity using Equation (4) and considering the values of J_ph_, J_o_, surface area (s), and incident light intensity (p), the optoelectronic photoelectrode exhibits optimal photoresponsivity values of 4.1 and 4.15 mA/W at 340 and 440 nm, respectively. This observation is consistent with expectations, as higher-energy photons with shorter wavelengths (such as those in the UV and blue regions) have the potential to generate a larger number of hot electrons. The higher photoresponsivity values indicate that the photoelectrode is more efficient in converting incident light into electrical signals when exposed to photons with higher energy or frequency [12,27,28,29].

(4) $$R=\frac{J_{ph}-J_{d}}{P\cdot S}$$

**Figure 7:**
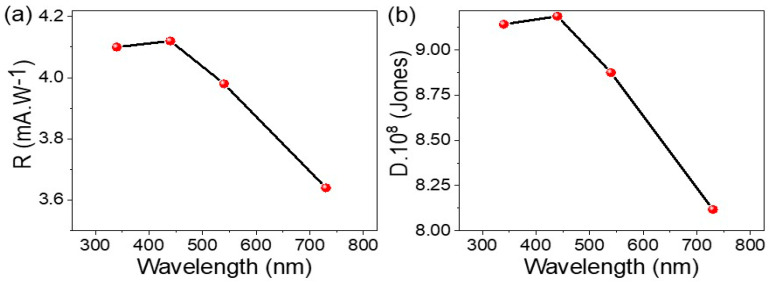
(**a**) The photoresponsivity and (**b**) the detectivity of MoO_2_-MoO_3_/Ppy core-shell nanocomposite optoelectronic electrode produced under the electrical measurements.

The efficiency of the photodetector can be further evaluated by estimating the detectivity (D) using Equation (5) and Figure 7b. The detectivity values for the MoO_2_-MoO_3_/Ppy core-shell nanocomposite photoelectrode are found to be 9.25 × 10^9^ and 9.30 × 10^8^ Jones at 340 and 440 nm, correspondingly. These values correspond to the high R observed in the previous analysis.

At 730 nm, the estimated detectivity value is 8.2 × 10^8^ Jones, suggesting that the optoelectronic photoelectrode retains its promising performance even at longer wavelengths.

Table 2 provides a comparison of these significant advancements achieved by the MoO_2_-MoO_3_/Ppy core-shell nanocomposite photoelectrode with other studies, highlighting its superior performance in terms of photoresponsivity. This further emphasizes the potential of this photoelectrode for various applications requiring accurate light detection and estimation.

(5) $$D=R\sqrt{S\;/2eJ_{o}\;}$$

## 4. Conclusions

In conclusion, a highly uniform spherical MoO_2_-MoO_3_/Ppy core-shell nanocomposite is synthesized as an optoelectronic photon sensing material that operates in the UV, Vis, and IR domains. The nanocomposite was prepared by oxidizing pyrrole with Na_2_MoO_4_, resulting in a morphology confirmed to be highly uniform and spherical through TEM, theoretical modeling, and SEM analyses. The nanocomposite demonstrates favorable optical characteristics, displaying a bandgap of 1.36 eV.

The optoelectronic photosensing performance of the nanocomposite was evaluated across the UV, Vis, and IR spectra, demonstrating high efficiency. The R values accurately reflect the generation of hot electrons in response to incident photons. Notably, the nanocomposite achieved an R value of 4.15 mA.W^−1^ at 440 nm, making it an effective choice for highly technological device applications.

The optoelectronic device sensitivity in the Vis region was confirmed by the D value of 9.30 × 10^8^ Jones at 440 nm. This indicates its ability to accurately detect light in this range. The remarkable stability of the device can be attributed to the highly stable MoO_2_-MoO_3_ oxide and Ppy polymer materials. This stability was demonstrated through reproducibility studies and current-voltage measurements conducted under various dark and light conditions. The exceptional stability and efficiency of this optoelectronic device make it well suited for light sensing applications in both industrial and commercial domains. Its performance opens up opportunities for advanced applications where reliable and precise light detection is required.

## Figures and Tables

**Table 1:** Functional groups positions of Ppy and MoO_2_-MoO_3_/Ppy from the FTIR spectroscopy.

Materials/Band Position (cm^−1^)		Characteristic Group
Ppy	MoO_2_-MoO_3_/Ppy	
3771	3754	N–H
	3419	O–H group
1621	1622	C=C quinoid
1545 and 1463	1548 and 1469	C=C benzene
1312	1309	C–N [22]
1177	1174	C–H
798, 1045, 910	1049 and 996	Ring vibration

**Table 2:** The MoO_2_-MoO_3_/Ppy photoresponsivity as compared to other studies.

Structure	Wavelength (nm)	Bais (V)	R (mAW^−1^)
GO/Cu_2_O [30]	300	2	0.5 × 10^−3^
PbI_2_-graphene [31]	550	2	NA
Graphene/P3HT [32]	325	1	NA
TiO_2_-PANI [33]	320	0	3 × 10^−3^
ZnO/RGO [34]	350	5	1.3 × 10^−3^
ZnO-CuO [35]	405	1	3 × 10^−3^
Graphene/GaN [36]	365	7	3 × 10^−3^
PbI_2_-graphene [31]	550	2	NA
CuO nanowires [37]	390	5	-
TiN/TiO_2_ [9]	550	5	-
ZnO/Cu_2_O [8]	350	2	4 × 10^−3^
Se/TiO_2_ [38]	450	1	5 × 10^−3^
PbI_2_-5%Ag [39]	532	6	NA
CuO/Si Nanowire [40]	405	0.2	3.8 × 10^−3^
MoO_2_-MoO_3_/Ppy (this work)	440	2	4.15
